# Dynamics of a Diffusive Predator-Prey Model with General Nonlinear Functional Response

**DOI:** 10.1155/2014/721403

**Published:** 2014-02-03

**Authors:** Wensheng Yang

**Affiliations:** School of Mathematics and Computer Science, Fujian Normal University, Fuzhou, Fujian 350007, China

## Abstract

We study a diffusive predator-prey model with nonconstant death rate and general nonlinear functional response. Firstly, stability analysis of the equilibrium for reduced ODE system is discussed. Secondly, sufficient and necessary conditions which guarantee the predator and the prey species to be permanent are obtained. Furthermore, sufficient conditions for the global asymptotical stability of the unique positive equilibrium of the system are derived by using the method of Lyapunov function. Finally, we show that there are no nontrivial steady state solutions for certain parameter configuration.

## 1. Introduction 

One of the dominant themes in both ecology and mathematical ecology is the dynamic relationship between predators and their prey due to its universal existence and importance in population dynamics. The investigations on predator-prey models are developed during these thirty years, and more realistic models are derived in view of laboratory experiments and observations (see [1–6] and the references therein).

In [7], Duque and Lizana considered the following reaction-diffusion predator-prey model with Holling type II functional response:

(1) $$\begin{array}{c} \frac{\partial N}{\partial t}=D_{1}\Delta N+ɛ\left(1-\frac{N}{K}\right)N-a\frac{PN}{\beta +N},\;x\in \Omega ,t>0,\\ \frac{\partial P}{\partial t}=D_{2}\Delta P-M\left(P\right)P+b\frac{NP}{\beta +N},\;x\in \Omega ,t>0,\\ \frac{\partial N}{\partial \upsilon }=\frac{\partial P}{\partial \upsilon }=0,\;x\in \partial \Omega ,t>0,\\ N\left(x,0\right)=\varphi_{1}\left(x\right)\geq 0,\;P\left(x,0\right)=\varphi_{2}\left(x\right)\geq 0,\;x\in \Omega , \end{array}$$

where *N*(*x*, *t*) and *P*(*x*, *t*) represent the population density of prey and predator at *x* ∈ *Ω* and at time *t*, respectively. The parameter *ɛ* > 0 is the specific growth rate of the prey in the absence of predation and without environment limitation; in the absence of predators, the prey population grows logistically to a carrying capacity *K* > 0; the functional response of the predator is a Holling type II function *a*(*PN*/(*β* + *N*)); *a*, *β*, and *b* are satiation coefficients or conversion rates. The specific mortality of predators in the absence of prey

(2) $$\begin{array}{c} M\left(P\right)=\frac{\gamma +\delta P}{1+P}=\delta +\frac{\gamma -\delta }{1+P},\;0<\gamma <\delta \end{array}$$

depends on the quantity of predator, *γ* is the mortality at low density, and *δ* is the maximal mortality with the natural assumption *γ* < *δ*. *D*_*i*_ > 0 are constants, *i* = 1,2, while Δ denotes the Laplace operator in *Ω* ⊂ *R* ^*n*^ with *Ω* bounded and connected. The advantage of the present model over the more often used models is that here the predator mortality is neither a constant nor an unbounded function, and still it is increasing with quantity.

In population dynamics, a functional response of the predator to the prey density refers to the change in the density of prey attached per unit time per predator as the prey density changes. The simplest functional response is a Lotka-Volterra function which is described as

(3) $$\begin{array}{c} \varphi \left(x\right)=ax,\;0\leq x\leq \frac{k}{a};\;\;\varphi \left(x\right)=k,\;x\geq \frac{k}{a} \end{array}$$

which is also called a Holling type I function. Michaelis and Menten proposed the response function

(4) $$\begin{array}{c} \varphi \left(x\right)=\frac{mx}{a+x} \end{array}$$

in studying enzymatic reactions. It is now referred to as a Michaelis-Menten function or a Holling type II function. However, *φ*(*x*) in Holling type I function and Holling type II function is monotonic in the first quadrant. It implies that, as the prey population increases, the consumption rate of prey per predator increases. But some experiments and observations indicate that a nonmonotonic response occurs at this level: when the nutrient concentration reaches a high level, an inhibitory effect on the specific growth rate may occur. To model such an inhibitory effect, Andrews [8] suggested a function

(5) $$\begin{array}{c} \varphi \left(x\right)=\frac{mx}{a+bx+x^{2}} \end{array}$$

called the Monod-Haldane function and also called a Holling type IV function. Sokol and Howell [9] proposed a simplified Holling type IV function of the form

(6) $$\begin{array}{c} \varphi \left(x\right)=\frac{mx}{a+x^{2}}. \end{array}$$

Motivated by the above question a functional response may be monotonic or nonmonotonic; we consider the following diffusive predator-prey model with general nonlinear functional response:

(7) $$\begin{array}{c} \frac{\partial N}{\partial t}=D_{1}\Delta N+ɛ\left(1-\frac{N}{K}\right)N-a\phi \left(N\right)P,\;x\in \Omega ,t>0,\\ \frac{\partial P}{\partial t}=D_{2}\Delta P-M\left(P\right)P+b\phi \left(N\right)P,\;x\in \Omega ,t>0,\\ \frac{\partial N}{\partial \upsilon }=\frac{\partial P}{\partial \upsilon }=0,\;x\in \partial \Omega ,t>0,\\ N\left(x,0\right)=\varphi_{1}\left(x\right)\geq 0,\;P\left(x,0\right)=\varphi_{2}\left(x\right)\geq 0,\;x\in \Omega , \end{array}$$

where *aϕ*(*N*) is a general functional response, and the function *ϕ*(*N*) is assumed to satisfy the following assumptions:

- *ϕ*(*N*) is of class *C* ^1^, *ϕ*(0) = 0;
- *N* ↦ *ϕ*(*N*)/*N* is decreasing on (0, +*∞*) and there exists a $\tilde{M}>0$ such that $\phi (N)/N\leq \tilde{M}$;
- there exists a *L* > 0 such that 0 ≤ *ϕ*(*N*) ≤ *L* for *N* ∈ [0, +*∞*);
- there exists a *H* > 0 such that |*ϕ*′(*N*)|≤*H* for *N* ∈ [0, +*∞*);
- there exists a *l* > 0 such that *ϕ*(*N*) is increasing for any *N* ∈ [0, *l*], where *K* < *l*.

Note that hypotheses (i)–(v) are satisfied if function *aϕ*(*N*) represents Holling type II functional response or Holling type IV functional response; that is, *aϕ*(*N*) = *aN*/(*β* + *N*) or *aϕ*(*N*) = *aN*/(*β* + *N* ^2^).

If *aϕ*(*N*) = *aN*/(*β* + *N*), system (7) reduces to the system (1). Duque and Lizana [7] obtained necessary and sufficient conditions under which the system (1) is permanent. To the best of the author knowledge, for the predator-prey system (7) with general functional response, whether one could obtain the sufficient and necessary conditions which insure the permanence of the system (7) or not is still an open problem.

The aim of this paper is, by further developing the analysis technique of Duque and Lizana [7], to obtain sufficient and necessary conditions which ensure the permanence of the system (7) and describe the global dynamic of the system (7). More concretely, we firstly discuss stability analysis of the equilibrium for reduced ODE system. Secondly, we obtain necessary and sufficient conditions under which the system is dissipative and permanent. Furthermore, we study the global stability of the nontrivial equilibrium when it is unique. Finally, we show that system (7) has no nontrivial steady state solutions for certain parameter configuration.

## 2. The Model without Diffusion 

For the reaction-diffusion predator-prey system (7), the reduced system is an ordinary differential equation of the form

(8) $$\begin{array}{c} \frac{dN}{dt}=ɛ\left(1-\frac{N}{K}\right)N-a\phi \left(N\right)P,\\ \frac{dP}{dt}=-M\left(P\right)P+b\phi \left(N\right)P. \end{array}$$

For simplicity, let us rewrite (8) as

(9) $$\begin{array}{c} \frac{dN}{dt}=a\phi \left(N\right)\left(f\left(N\right)-P\right),\\ \frac{dP}{dt}=P\left(-M\left(P\right)+b\phi \left(N\right)\right), \end{array}$$

where

(10) $$\begin{array}{c} f\left(N\right)=\frac{ɛ\left(1-N/K\right)N}{\left(a\phi \left(N\right)\right)}. \end{array}$$

The equilibria of (9) consist of two trivial critical points *E*_1_ = (0,0) and *E*_2_ = (*K*, 0) on the boundary of *Ω* = {(*N*, *P*) : *N* ≥ 0, *P* ≥ 0} and a set of nontrivial critical points obtained as the intersection of the curves

(11) $$\begin{array}{c} P=f\left(N\right),\;\;M\left(P\right)=b\phi \left(N\right). \end{array}$$

It is well known that persistence implies the existence of a positive equilibrium; see, for instance, [10]. From Theorem 8 in Section 3, one can easily know that system (9) has a positive equilibrium point *E*_3_ = (*N*_0_, *P*_0_), if −*γ* + *bϕ*(*K*) > 0, where

(12) $$\begin{array}{c} P_{0}=f\left(N_{0}\right),\;\;M\left(P_{0}\right)=b\phi \left(N_{0}\right). \end{array}$$

The Jacobian matrix of system (9) at *E*_1_ = (0,0) is

(13) $$\begin{array}{c} J_{E_{1}}=\left(\begin{array}{cc} ɛ & 0\\ 0 & -\gamma \end{array}\right), \end{array}$$

and then it is clear that the Jacobian matrix *J*_*E*_1__ has two eigenvalues *λ*_1_ = *ɛ*, *λ*_2_ = −*γ*. Hence, the equilibrium *E*_1_ = (0,0) is a saddle point and the stable and unstable manifolds lie on the *P*-axis and *N*-axis, respectively.

The Jacobian matrix of system (9) at *E*_2_ = (*K*, 0) is

(14) $$\begin{array}{c} J_{E_{2}}=\left(\begin{array}{cc} -ɛ & -a\phi \left(K\right)\\ 0 & -\gamma +b\phi \left(K\right) \end{array}\right), \end{array}$$

and then it is clear that the Jacobian matrix *J*_*E*_2__ has two eigenvalues *λ*_1_ = −*ɛ*, *λ*_2_ = −*γ* + *bϕ*(*K*). Hence the equilibrium *E*_2_ = (*K*, 0) is a saddle point, if −*γ* + *bϕ*(*K*) > 0, and it is locally asymptotically stable when −*γ* + *bϕ*(*K*) ≤ 0.

The Jacobian matrix of system (9) at *E*_3_ = (*N*_0_, *P*_0_) is

(15) $$\begin{array}{c} J=\left(\begin{array}{cc} a\phi \left(N_{0}\right)f^{\prime }\left(N_{0}\right) & -a\phi \left(N_{0}\right)\\ bP_{0}\phi^{\prime }\left(N_{0}\right) & -P_{0}M^{\prime }\left(P_{0}\right) \end{array}\right), \end{array}$$

and the eigenvalues of the matrix *J* are the roots of the polynomial

(16) $$\begin{array}{c} \det\left[J-\rho I\right]=\rho^{2}-\text{trace}\;J\rho +\det J=0, \end{array}$$

where

(17) $$\begin{array}{c} \text{trace}\;J=J_{11}+J_{22},\;\;\det J=J_{11}J_{22}-J_{12}J_{21},\\ J_{11}=a\phi \left(N_{0}\right)f^{\prime }\left(N_{0}\right),\;\;J_{22}=-P_{0}M^{\prime }\left(P_{0}\right),\\ J_{12}=-a\phi \left(N_{0}\right),\;\;J_{21}=bP_{0}\phi^{\prime }\left(N_{0}\right). \end{array}$$

The following result holds.

**Proposition**

If *N*_0_ ∈ [*K*/2, *K*], then the equilibrium *E*_3_ = (*N*_0_, *P*_0_) is locally asymptotically stable with respect to system (9).

**Proof**

Taking into account that *N*_0_ ∈ [*K*/2, *K*], the assumptions (i)–(v), and (2), it follows that

(18) f′(N0)=a(ɛ−2ɛKN0)ϕ(N0)−aɛ(1−N0K)N0ϕ′(N0)≤0, M′(P0)>0,

which in turn implies that trace*J* = *J*_11_ + *J*_22_ = *aϕ*(*N*_0_)*f*′(*N*_0_) − *P*_0_ *M*′(*P*_0_) < 0. Moreover, since *J*_11_, *J*_22_, *J*_12_ < 0 and *J*_21_ > 0, it follows that det⁡(*J*) > 0, which completes the proof.

## 3. Extinction of the Predator and Permanence 

In this section, we will show that any nonnegative solution (*N*(*x*, *t*), *P*(*x*, *t*)) of (7) lies in a certain bounded region as *t* → *∞* for all *x* ∈ *Ω*. We first show a well-known conclusion on the Logistic equation.

**Lemma (see [6])**

Assume that *u*(*x*, *t*) is defined by

(19) $$\begin{array}{c} \frac{\partial u}{\partial t}=d_{1}\Delta u+ru\left(1-\frac{u}{K}\right),\;x\in \Omega ,t>0,\\ \frac{\partial u}{\partial \upsilon }=0,\;x\in \partial \Omega ,t>0,\\ u\left(x,0\right)=u_{0}\left(x\right)>0,\;x\in \Omega , \end{array}$$

then lim⁡_*t*→*∞*_ *u*(*x*, *t*) = *K*.

**Theorem 3**

All the solutions of (7) are nonnegative and defined for all *t* > 0. Furthermore, the nonnegative solution (*N*(*x*, *t*), *P*(*x*, *t*)) of (7) satisfies

(20) $$\begin{array}{c} \underset{t\to +\infty }{\mathrm{limsup}\;}\underset{x\in \bar{\Omega }}{\max}\;N\left(x,t\right)\leq K,\\ \underset{t\to +\infty }{\mathrm{limsup}\;}\underset{x\in \bar{\Omega }}{\max}\;P\left(x,t\right)\leq \frac{ɛ\left(K+1\right)}{a\phi \left(K+1\right)}. \end{array}$$

**Proof**

The nonnegativity of the solutions of (7) is clear since the initial value is nonnegative. We only consider the latter of the theorem.

From the first equation of system (7), we have

(21) ∂N∂t=D1ΔN+ɛ(1−NK)N−aϕ(N)P≤D1ΔN+ɛ(1−NK)N,

then from comparison principle of parabolic equations and Lemma 2, for an arbitrary *η* > 0, there exists *T*_*η*_(>0) such that, for any *t* > *T*_*η*_,

(22) $$\begin{array}{c} N\left(x,t\right)\leq K+\eta . \end{array}$$

This implies

(23) $$\begin{array}{c} \underset{t\to +\infty }{\mathrm{limsup}\;}\underset{x\in \bar{\Omega }}{\max}\;N\left(x,t\right)\leq K. \end{array}$$

Now, let us prove the boundedness of *P*(*x*, *t*). Taking into account the second equation of system (7) and the fact that *γ* ≤ *M*(*P*) ≤ *δ*, we get

(24) ∂P∂t−D2ΔP=−M(P)P+bϕ(N)P,≤−γP+bϕ(N)P.

Let us show that there cannot exist a *T* = *T*(*M*_0_) > 0 such that

(25) $$\begin{array}{c} P\left(x,t\right)>M_{0},\;\forall t\geq T,x\in \bar{\Omega }, \end{array}$$

where

(26) $$\begin{array}{c} M_{0}=\frac{ɛ\left(K+1\right)}{a\phi \left(K+1\right)}. \end{array}$$

Indeed, assume that (25) is true. Choosing *η* = 1 and taking into account that

(27) $$\begin{array}{c} N\left(x,t\right)\leq K+1,\;t\geq T_{\eta },x\in \bar{\Omega }, \end{array}$$

we get that

(28) $$\begin{array}{c} \frac{a\phi \left(N\right)}{N}P\geq \frac{a\phi \left(K+1\right)}{K+1}P\geq ɛ,\;\forall t\geq T_{*},x\in \bar{\Omega }, \end{array}$$

where *T*_∗_ = max⁡{*T*_*η*_, *T*}. Then, from (28) and the first equation of system (7), we obtain

(29) ∂N∂t−D1ΔN=ɛ(1−NK)N−aϕ(N)P=−ɛKN2+N(ɛ−aϕ(N)NP)≤−ɛKN2, ∀t≥T∗.

This immediately implies that

(30) $$\begin{array}{c} 0<N\left(x,t\right)\leq z\left(t\right)⟶0,\;t⟶+\infty ,x\in \bar{\Omega }, \end{array}$$

where *z*(*t*) is the solution of the equation

(31) $$\begin{array}{c} z^{\prime }\left(t\right)=-\frac{ɛ}{K}z^{2}\left(t\right),\;\;z\left(T_{*}\right)=\underset{x\in \bar{\Omega }}{\max\;}N\left(x,T_{*}\right). \end{array}$$

Since

(32) $$\begin{array}{c} b\phi \left(N\right)=b\frac{\phi \left(N\right)}{N}N, \end{array}$$

(33) $$\begin{array}{c} \underset{N\to 0}{\lim}\frac{\phi \left(N\right)}{N}=\underset{N\to 0}{\lim}\phi^{\prime }\left(N\right)=\phi^{\prime }\left(0\right). \end{array}$$

Having in mind that (30) and (33) hold, we get that

(34) $$\begin{array}{c} \underset{N\to 0}{\lim}\frac{\phi \left(N\right)}{N}N=0. \end{array}$$

From (34), we can easily obtain that there exists *t*_0_ ≥ *T*_∗_ such that

(35) $$\begin{array}{c} b\phi \left(N\right)=b\frac{\phi \left(N\right)}{N}N\leq \frac{\gamma }{2},\;\forall t\geq t_{0},x\in \bar{\Omega }. \end{array}$$

Now, taking into account the second equation of system (7) and (35), we get

(36) ∂P∂t−D2ΔP=−M(P)P+bϕ(N)P≤−γP+bϕ(N)P≤(−γ+γ2)P≤−γ2P.

By using the comparison principle, we obtain that *P*(*x*, *t*) → 0, *t* → *∞*, which is a contradiction.

Let us define the function Φ(*x*, *t*) = *P*(*x*, *t*) − *M*_0_, $x\in \bar{\Omega }$ and *t* > 0. We have to consider two possibilities. If there is some *t*_0_ such that Φ(*x*, *t*) ≠ 0 for any *t* > *t*_0_, we say that the zeroes of Φ are bounded. If this is not true we say that the zeroes are unbounded; in this case, Φ(*x*, *t*) = 0 for a sequence of *t*_*n*_ tending to +*∞* as *n* → *∞*.

If the zeroes of Φ(*x*, *t*) are bounded, then 0 < *P*(*x*, *t*) ≤ *M*_0_ for any *t* ≥ *t*_0_.

If the zeroes are unbounded, the *t*-axis is divided by the zeroes into a sequence of intervals *J*_*n*_, *n* ≥ 1. In each, such interval Φ(*x*, *t*) is of constant sign. Let us assume that Φ(*x*, *t*) ≥ 0 for *t* ∈ *J*_*n*_, *n* = 1,3, 5,…. Since *P*(*x*, *t*) ≥ *M*_0_ on the intervals *J*_2*n*−1_, arguing as in the proof that (25) is not possible, we obtain that

(37) $$\begin{array}{c} \frac{\partial P}{\partial t}-D_{2}\Delta P\leq -\frac{\gamma }{2}P \end{array}$$

for *t* ∈ *J*_2*n*−1_ and *n* which is large enough, and $x\in \bar{\Omega }$. So, by the comparison principle, *P*(*x*, *t*) ≤ *z*(*t*) for *t* ∈ *J*_2*n*−1_, where *z* is the solution of the equation *z*′(*t*) = −(*γ*/2)*z*(*t*), with a suitable initial condition. Thus,

(38) $$\begin{array}{c} P\left(x,t\right)\leq z\left(t\right)\leq z\left(t_{2n-1}\right)=M_{0},\;\forall t\in J_{2n-1},x\in \bar{\Omega }, \end{array}$$

and *n* which is large enough. This contradiction completes the proof of our claim.

**Remark 4**

An immediate consequence of the proof of the former result is that for a given η* > 0,

(39) Λ={(N,P)∈R2:N∈[0,K+η∗],P∈[0,ɛ(K+1)aϕ(K+1)+η∗]}

is an absorbing set for system (7). Hence, system (7) is dissipative.

Now, we will summarize some facts contained in Hale and Waltman studies, see [11], about the permanence for abstract dynamical systems.

Suppose that *Ω* is a complete metric space with *Ω* = *Ω*_0_ ∪ ∂*Ω*_0_ for an open set *Ω*_0_, where ∂*Ω*_0_ is the boundary of the set *Ω*_0_. We will typically choose *Ω*_0_ to be the positive cone in an ordered Banach space. A flow or semiflow on *Ω* under which *Ω*_0_ and ∂*Ω*_0_ are forward invariant is said to be permanent if it is dissipative and if there is a number *η* > 0 such that any trajectory starting in *Ω*_0_ will be at least a distance *η* from ∂*Ω*_0_ for all sufficiently large *t*. To state a theorem implying permanence, we need a few definitions. An invariant set *M* for the flow or semiflow is said to be isolated if it has a neighborhood *U* such that *M* is the maximal invariant subset of *U*. Let *ω*(∂*Ω*_0_)⊂∂*Ω*_0_ denote the union of the sets *ω*(*u*) over *u* ∈ ∂*Ω*_0_. This differs from the standard definition of the *ω*-limit set of a set but it is more convenient for our purposes; see [10] for a discussion. The set *ω*(*Ω*_0_) is said to be isolated if it has a covering *M* = ∪_*k*=1_^*n*^ *M*_*k*_ of pairwise disjoint, both sets *M*_*k*_ which are isolated and invariant with respect to the flow or semiflow both on ∂*Ω*_0_ and on *Ω* = *Ω*_0_ ∪ ∂*Ω*_0_. The covering *M* is then called an isolated covering. Suppose *N*_1_ and *N*_2_ are isolated invariant sets (not necessarily distinct). The set *N*_1_ is said to be chained to *N*_2_ (denoted by *N*_1_ → *N*_2_) if there exists *u* ∉ *N*_1_ ∪ *N*_2_ with *u* ∈ *W* ^*u*^(*N*_1_)∩*W* ^*s*^(*N*_2_). As usual, *W* ^*u*^ and *W* ^*s*^ denote the unstable and stable manifolds, respectively. A finite sequence *N*_1_, *N*_2_,…, *N*_*k*_ of isolated invariant sets is a chain if *N*_1_ → *N*_2_ → *N*_3_ → ⋯→*N*_*k*_. This is possible for *k* = 1 if *N*_1_ → *N*_1_. The chain is called a cycle if *N*_*k*_ = *N*_1_. The set *ω*(∂*Ω*_0_) is said to be acyclic if there exists an isolated covering ∪_*k*=1_^*n*^ *M*_*k*_ such that no subset of {*M*_*k*_} is a cycle. We now state a lemma that can be used to establish permanence.

**Lemma 5 (see [11])**

Suppose that *Ω* is a complete metric space with *Ω* = *Ω*_0_ ∪ ∂*Ω*_0_, where *Ω*_0_ is open. Suppose that a semiflow on *Ω* leaves both *Ω*_0_ and ∂*Ω*_0_ forward invariant, maps bounded sets in *Ω* to precompact sets for *t* > 0, and is dissipative. If in addition

1. *ω*(∂*Ω*_0_) is isolated and acyclic,
2. *W* ^*s*^(*M*_*k*_)∩*Ω*_0_ = *∅* for all *k*, where ∪_*k*=1_^*n*^ *M*_*k*_ is the isolated covering used in the definition of acyclicity of *ω*(∂*Ω*_0_),

then the semiflow is permanent; that is, there exists *η* > 0 such that any trajectory with initial data in *Ω*_0_ will be bounded away from ∂*Ω*_0_ by a distance greater than *η* for *t* which is sufficiently large.

Now, we show that system (7) is permanent. From the viewpoint of biology, this implies that the two species of prey and predator will always coexist at any time and any location of the inhabit domain, no matter what their diffusion coefficients are. First, we identify a less interesting situation where the predator cannot survive.

**Proposition 6**

If −*γ* + *bϕ*(*K*) ≤ 0, then *E*_2_ = (*K*, 0) is globally asymptotically stable with respect to nonnegative initial functions.

**Proof**

Let us assume that −*γ* + *bϕ*(*K*) ≤ 0, we can easily obtain that, for small enough ε* > 0,

(40) $$\begin{array}{c} -\gamma +b\phi \left(K+\varepsilon^{*}\right)\leq 0. \end{array}$$

From Theorem 3, it follows that, for the above ε* > 0, there exists a *T* > 0, such that 0 ≤ *N*(*x*, *t*) ≤ *K* + ε*, for all *t* ≥ *T*, *x* ∈ *Ω*.

Therefore, from the second equation of system (7), we obtain that

(41) ∂P∂t−D2ΔP=−M(P)P+bϕ(N)P=[−γ+bϕ(N)−(δ−γ)P1+P]P≤[−γ+bϕ(K+ε∗)]P−(δ−γ)P21+P

for all *t* ≥ *T*. Invoking again the comparison principle, we get that *P*(*x*, *t*) ≤ *v*(*t*) for any *t* ≥ *T*, where *v*(*t*) is the solution of the equation *v*′ = *H*_2_(*v*)*v*, with $v(T)=\max_{x\in \bar{\Omega }}P(x,T)>0$, where

(42) H2(v)=[−γ+bϕ(K+ε∗)−(δ−γ)v1+v]<0,∀t≥T.

This implies that *P*(*x*, *t*) → 0 as *t* → *∞*. Henceforth, we may assume that, under the hypothesis of our claim, for any ε* > 0, 0 ≤ *P*(*t*) ≤ ε*, for all *t* ≥ *T*. Now, from the first equation of system (7), we obtain

(43) ∂N∂t−D1ΔN=ɛ(1−NK)N−aϕ(N)P≥ɛ(1−NK)N−aϕ(N)ε∗≥ɛ(1−NK)N−aLε∗.

By the comparison principle, we get that *N*(*x*, *t*) ≥ *u*(*t*) for any *t* ≥ *T*, where *u*(*t*) is the solution of the equation *u*′ = *ɛ*(1 − *u*/*K*)*u* − aLε*. Finally, our assertion follows from the arbitrariness of ε* and the fact that the solutions with positive initial data of the equation *u*′ = *ɛ*(1 − *u*/*K*)*u* tend exponentially to *u* = *K*.

From the above discussion and Theorem 3, we obtain that *E*_2_ = (*K*, 0) is globally asymptotically stable.

**Remark 7**

If *ϕ*(*N*) = *N*/(*β* + *N*), system (7) reduces to system (1). So, it is shown that our result supplements and covers the Proposition 4.1 of Duque and Lizana's paper [7].

**Theorem 8**

System (7) is permanent if and only if

(44) $$\begin{array}{c} -\gamma +b\phi \left(K\right)>0. \end{array}$$

**Proof**

Let us set *F* = (*F*_1_, *F*_2_), *U* = (*N*, *P*), and *D* = diag⁡[*D*_1_, *D*_2_], where

(45) $$\begin{array}{c} F_{1}\left(N,P\right)=ɛ\left(1-\frac{N}{K}\right)N-a\phi \left(N\right)P,\\ F_{2}\left(N,P\right)=-M\left(P\right)P+b\phi \left(N\right)P. \end{array}$$

Henceforth, considering also an initial condition, system (7) can be rewritten as

(46) $$\begin{array}{c} \frac{\partial U}{\partial t}\left(x,t\right)=D\Delta U\left(x,t\right)+F\left(U\right),\;x\in \Omega ,t>0,\\ \frac{\partial U}{\partial \upsilon }\left(x,t\right)=0,\;x\in \partial \Omega ,t>0,\\ U\left(x,0\right)=\varphi \left(x\right)=\left(\varphi_{1}\left(x\right),\varphi_{2}\left(x\right)\right),\;x\in \Omega . \end{array}$$

Denote $\Omega_{0}=\{\varphi \in X_{\Lambda }:\varphi (x)>0,x\in \bar{\Omega }\}$, where *X*_Λ_ = *Ω*_0_ ∪ ∂*Ω*_0_. Assume that operator *S*(*t*) is compact for *t* > 0, which is defined by [*S*(*t*)*φ*](*x*) = *U*(*x*, *t*; *φ*), where *U*(*x*, *t*; *φ*) is the classical solution of system (7). Moreover, from Theorem 3, it follows that the semiflow {*S*(*t*)}_*t*≥0_ is pointwise dissipative.

A direct application of the maximum principle shows that *S*(*t*) is positively invariant on ∂*Ω*_0_, which in turn implies that *S*(*t*) is positively invariant on *Ω*_0_.

Now, let us show that *ω*(∂*Ω*_0_) = {*E*_1_, *E*_2_}. First, we consider

(47) $$\begin{array}{c} \frac{\partial N}{\partial t}=D_{1}\Delta N+ɛ\left(1-\frac{N}{K}\right)N,\;x\in \Omega ,t>0,\\ \frac{\partial N}{\partial \upsilon }=0,\;x\in \partial \Omega ,t>0,\\ N\left(x,0\right)=\varphi_{1}\left(x\right)>0,\;x\in \Omega . \end{array}$$

There is no loss of generality assuming that *φ*_1_(*x*) > 0 for all $x\in \bar{\Omega }$. Indeed, if *φ*_1_(*x*) ≥ 0 for all $x\in \bar{\Omega }$ with *φ*_1_(*x*)≢0 and applying the strong maximum principle, we obtain that *N*(*x*, *t*) > 0 for all *t* > 0 and $x\in \bar{\Omega }$. One could change the origin of time to a positive time *t*_1_ and choose *φ*_1_(*x*) = *N*(*x*, *t*_1_).

Let *u*(*t*) and *ω*(*t*) be the solutions of the equation *z*′ = *ɛ*(1 − *z*/*K*)*z* such that $u(0)=m=\min_{x\in \bar{\Omega }}\varphi_{1}(x)>0$ and $\omega (0)=M=\max_{x\in \bar{\Omega }}\varphi_{1}(x)>0$, respectively. From the comparison principle, we obtain

(48) $$\begin{array}{c} u\left(t\right)\leq N\left(x,t\right)\leq \omega \left(t\right),\;t>0,x\in \bar{\Omega }. \end{array}$$

Taking into account that *z*(*t*) = *K* attracts any positive solution, we conclude that *N*(*x*, *t*) → *K* as *t* → *∞*.

Let us now consider

(49) $$\begin{array}{c} \frac{\partial P}{\partial t}=D_{2}\Delta P-M\left(P\right)P,\;x\in \Omega ,t>0,\\ \frac{\partial P}{\partial \upsilon }=0,\;x\in \partial \Omega ,t>0,\\ P\left(x,0\right)=\varphi_{2}\left(x\right)\geq 0,\;x\in \Omega . \end{array}$$

Since *M*(*P*) ≥ *γ*, we get that

(50) $$\begin{array}{c} \frac{\partial P}{\partial t}-D_{2}\Delta P=-M\left(P\right)P\leq -\gamma P; \end{array}$$

by the comparison principle, we obtain that *P*(*x*, *t*) → 0 as *t* → *∞*. Henceforth, we conclude that *ω*(∂*Ω*_0_) = {*E*_1_, *E*_2_} and it is isolated and acyclic. By choosing *M*_1_ = *E*_1_ and *M*_2_ = *E*_2_, then *M* = *M*_1_ ∪ *M*_2_ is the covering required by Lemma 2.

Taking into account that *ω*(∂*Ω*_0_) is positively invariant and the fact that the stable and unstable manifolds of the equilibrium *E*_1_ lie on *ω*(∂*Ω*_0_), we obtain that *W* ^*s*^(*E*_1_)∩*Ω*_0_ = *∅*. In order to show that *W* ^*s*^(*E*_2_)∩*Ω*_0_ = *∅*, let us linearize (7) about *E*_2_, obtaining the following system:

(51) $$\begin{array}{c} \frac{\partial N}{\partial t}=D_{1}\Delta N-ɛN-a\phi \left(K\right)P,\\ \frac{\partial P}{\partial t}=D_{2}\Delta P+\left(-\gamma +b\phi \left(K\right)\right)P. \end{array}$$

From (44) and by using comparison techniques, we obtain the unstable manifold to *E*_2_ point into the region *Ω*_0_. Moreover, from the above performed computations we know that the stable manifold corresponding to the equilibrium *E*_2_ lies on ∂*Ω*_0_. This implies that *W* ^*s*^(*E*_2_)∩*Ω*_0_ = *∅*.

Since all hypotheses of Lemma 5 are fulfilled, we may conclude that system (7) is permanent.

Permanence implies that condition (44) is an immediate consequence of Theorem 3. This concludes the proof of our claim.

**Remark 9**

From Theorem 8, we obtain that system (7) with nonmonotonic functional response is also permanent if and only if (44) holds. That is, there exist positive constants *q*, *Q*, *w*, and *W* such that *q* ≤ *N*(*x*, *t*) ≤ *Q*, *w* ≤ *N*(*x*, *t*) ≤ *W* as time converges to infinity. If *ϕ*(*N*) = *N*/(*β* + *N*), system (7) reduces to system (1). So, it is shown that our result supplements and covers Theorem 4.1 of Duque and Lizana's paper [7].

**Remark 10**

We can easily obtain that in case the condition (44) is not satisfied, system (7) has no nontrivial equilibria. This fact is not surprising, it is well-known that persistence implies the existence of a positive equilibrium; see, for instance [10].

**Remark 11**

From Theorem 8, it is shown that diffusion has no influence on the permanence of system (7).

## 4. Global Stability of the Nontrivial Equilibrium 

Hereafter in this paper, we restrict our attention to the case −*γ* + *bϕ*(*K*) > 0, in this case system (9) has a nontrivial equilibrium point. It is obvious that the equilibria of system (9) are solutions of (7). We will focus our attention on the nontrivial equilibrium *U*_0_ = (*N*_0_, *P*_0_) of the system (9). Concretely, in this section, we will analyze the local and the global stability of nontrivial equilibrium of (7).

Let us first briefly study the local stability of the homogeneous stationary solution *U*_0_ of (7) via the linearized stability analysis (see [12, pp. 68–70]). Setting *W* = *U* − *U*_0_ and recalling that

(52) $$\begin{array}{c} A=F^{\prime }\left(U_{0}\right)=\left(\begin{array}{cc} a\phi \left(N_{0}\right)f^{\prime }\left(N_{0}\right) & -a\phi \left(N_{0}\right)\\ bP_{0}\phi^{\prime }\left(N_{0}\right) & -P_{0}M^{\prime }\left(P_{0}\right) \end{array}\right), \end{array}$$

the linearized system of the reaction-diffusion equation (7) about *U*_0_ is given by

(53) $$\begin{array}{c} \frac{\partial W}{\partial t}=D\Delta W+AW,\;x\in \Omega ,t>0,\\ \frac{\partial W}{\partial \upsilon }\left(x,t\right)=0,\;x\in \partial \Omega ,t>0. \end{array}$$

The trivial solution *W* = 0 is asymptotically stable if and only if every solution of (53) decays to zero as *t* → *∞*. Following [12, pp. 68–70], we obtain that the trivial solution *W* = 0 of (53) is asymptotically stable if and only if each solution of the system

(54) $$\begin{array}{c} \frac{ds_{j}}{dt}=B_{j}s_{j}=\left(A-\lambda_{j}D\right)s_{j} \end{array}$$

tends to zero as *t* → *∞* for each *j* ≥ 0. *λ*_*j*_ denotes the *j*th eigenvalue corresponding to the *j*th eigenfunction *ϕ*_*j*_(*x*) of the operator −Δ on *Ω* with no-flux boundary conditions. Let us recall that the eigenvalues {*λ*_*j*_}_*j*=0_^*∞*^ satisfy the following inequality:

(55) $$\begin{array}{c} 0=\lambda_{0}<\lambda_{1}<\lambda_{2}<\cdots , \end{array}$$

where

(56) $$\begin{array}{c} \lambda_{j}=\frac{\int_{\Omega }{\left|\nabla \phi_{j}\right|}^{2}dx}{\int_{\Omega }\phi_{j}^{2}dx}>0,\;\forall j\geq 1. \end{array}$$

See, for instance, [13, pp. 205–208].

Henceforth, the asymptotic stability of *W* = 0 is equivalent to that each matrix *B*_*j*_ has two eigenvalues with negative real parts for all *j* ≥ 0. And the eigenvalues of the matrix *B*_*j*_ are the roots of the polynomial

(57) $$\begin{array}{c} \det\left[B_{j}-\rho I\right]=\rho^{2}-\text{trace}\;B_{j}\rho +\det B_{j}=0, \end{array}$$

where

(58) $$\begin{array}{c} \text{trace}\;B_{j}=A_{11}+A_{22}-\lambda_{j}\left(D_{1}+D_{2}\right),\\ \det B_{j}=\left(A_{11}-\lambda_{j}D_{1}\right)\left(A_{22}-\lambda_{j}D_{2}\right)-A_{12}A_{21},\\ A_{11}=a\phi \left(N_{0}\right)f^{\prime }\left(N_{0}\right),\;\;A_{22}=-P_{0}M^{\prime }\left(P_{0}\right),\\ A_{12}=-a\phi \left(N_{0}\right),\;\;A_{21}=bP_{0}\phi^{\prime }\left(N_{0}\right). \end{array}$$

The following result holds.

**Proposition 12**

If *N*_0_ ∈ [*K*/2, *K*], then the equilibrium *U*_0_ is locally asymptotically stable with respect to system (7).

**Proof**

Taking into account that *N*_0_ ∈ [*K*/2, *K*], the assumptions (i)–(v), and (2), it follows that

(59) f′(N0)=a(ɛ−2ɛKN0)ϕ(N0)−aɛ(1−N0K)N0ϕ′(N0)≤0, M′(P0)>0,

which in turn implies that trace*B*_*j*_ = *A*_11_ + *A*_22_ − *λ*_*j*_(*D*_1_ + *D*_2_) = *aϕ*(*N*_0_)*f*′(*N*_0_) − *P*_0_ *M*′(*P*_0_) − *λ*_*j*_(*D*_1_ + *D*_2_) < 0. Moreover, since *A*_11_, *A*_22_, *A*_12_ < 0 and *A*_21_ > 0, it follows that det⁡*B*_*j*_ = (*A*_11_ − *λ*_*j*_ *D*_1_)(*A*_22_ − *λ*_*j*_ *D*_2_) − *A*_12_ *A*_21_ > 0, which completes the proof.

**Remark 13**

From Propositions 1 and 12, we can easily obtain that diffusion has no influence on the stability of the equilibrium *U*_0_.

Now, we will focus on the global stability of *U*_0_. Biologically, our statement of the global stability of *U*_0_ means that no matter the two species diffuse, they will be spatially homogeneously distributed as time converges to infinity.

**Theorem 14**

Assume that

(60) $$\begin{array}{c} \frac{\left(q+N_{0}\right)ɛ}{KQ}-\frac{ɛ}{q}-\frac{a\tilde{M}}{2}-\frac{bP_{0}H}{2w}>0,\\ \frac{\gamma }{W}-\frac{bL}{w}-\frac{bP_{0}H}{2w}-\frac{a\tilde{M}}{2}>0 \end{array}$$

hold; then *U*_0_ is globally asymptotically stable.

**Proof**

Let us define the following function:

(61) $$\begin{array}{c} E\left(t\right)=\int_{\Omega }\left[N-N_{0}-N_{0}\ln\left(\frac{N}{N_{0}}\right)+P-P_{0}-P_{0}\ln\left(\frac{P}{P_{0}}\right)\right]dx. \end{array}$$

It is easy to show that *E*(*t*) ≥ 0 for all *t* ≥ 0. Differentiating *E*(*t*) along the solutions of the system (7), we obtain

(62) E′(t)=∫Ω[(1−N0N)Nt+(1−P0P)Pt]dx≤−D1N0∫Ω|∇N|2N2dx−D2P0∫Ω|∇P|2P2dx −((q+N0)ɛKQ−ɛq−aM~2−bP0H2w) ×∫Ω(N−N0)2dx −(γW−bLw−bP0H2w−aM~2)∫Ω(P−P0)2dx.

From (60), we can easily obtain that

(63) $$\begin{array}{c} \frac{dE\left(t\right)}{dt}=\int_{\Omega }\left[\left(1-\frac{N_{0}}{N}\right)N_{t}+\left(1-\frac{P_{0}}{P}\right)P_{t}\right]dx<0. \end{array}$$

Thus, *E*(*t*) satisfies Lyapunov's asymptotic stability theorem, and the positive equilibrium *U*_0_ of system (7) is globally stable. This completes the proof.

## 5. Nonexistence of Nonconstant Positive Steady States 

The main purpose of this section is to prove that, under certain restriction on the coefficients, (7) has no nontrivial positive steady state solutions. To accomplish this goal, first, we will obtain a priori bounds for the positive steady states of system (7). The corresponding steady state problem of (7) is the elliptic system

(64) $$\begin{array}{c} -D_{1}\Delta N=ɛ\left(1-\frac{N}{K}\right)N-a\phi \left(N\right)P,\;x\in \Omega ,\\ -D_{2}\Delta P=-M\left(P\right)P+b\phi \left(N\right)P,\;x\in \Omega ,\\ \frac{\partial N}{\partial \upsilon }=\frac{\partial P}{\partial \upsilon }=0,\;x\in \partial \Omega . \end{array}$$

Now let us state the following result that we will need in what follows.

**Lemma 15 (see [14])**

Suppose that $h\in C(\bar{\Omega }\times R)$; then the following results hold.

1. If $\omega \in C^{2}(\Omega )\cap C^{1}(\bar{\Omega })$ satisfies Δ*ω*(*x*) + *g*(*x*, *ω*(*x*)) ≥ 0 for *x* ∈ *Ω*, ∂*ω*/∂*υ* ≤ 0 for *x* ∈ ∂*Ω*, and $\omega (x_{0})=\max_{x\in \bar{\Omega }}\omega$, then *g*(*x*_0_, *ω*(*x*_0_)) ≥ 0.
2. If $\omega \in C^{2}(\Omega )\cap C^{1}(\bar{\Omega })$ satisfies Δ*ω*(*x*) + *g*(*x*, *ω*(*x*)) ≤ 0 for *x* ∈ *Ω*, ∂*ω*/∂*υ* ≥ 0 for *x* ∈ ∂*Ω*, and $\omega (x_{0})=\min_{x\in \bar{\Omega }}\omega$, then *g*(*x*_0_, *ω*(*x*_0_)) ≤ 0.

The following result gives an upper estimate for classical solutions of (64), where classical solution means solution in $C^{2}(\Omega )\cap C^{1}(\bar{\Omega })$. Let us point out that the same upper bounds that we obtained for the dissipativity are valid for the elliptic problem. But, by using Lemma 15, we will obtain the sharpest bounds.

**Proposition 16**

Let *K*, *b* such that *γ* < *bϕ*(*K*) < *δ*; then for any positive classical solution (*N*, *P*) of system (64), the following estimates hold:

(65) $$\begin{array}{c} \underset{x\in \bar{\Omega }}{\max}\;N\left(x\right)\leq K,\;\;\underset{x\in \bar{\Omega }}{\max}\;P\left(x\right)\leq \frac{b\phi \left(K\right)-\gamma }{\delta -b\phi \left(K\right)}. \end{array}$$

**Proof**

Let us set *ψ*_1_ = *D*_1_ *N*. From the first equation of system (64), we obtain that

(66) $$\begin{array}{c} \Delta \psi_{1}+\left[ɛ\left(1-\frac{N}{K}\right)-a\frac{\phi \left(N\right)}{N}P\right]N=0,\;x\in \Omega ,\\ \frac{\partial \psi_{1}}{\partial \upsilon }=0,\;x\in \partial \Omega . \end{array}$$

Let $x_{0}\in \bar{\Omega }$ be such that $\psi_{1}(x_{0})=\max_{x\in \bar{\Omega }}\psi_{1}$. Then by Lemma 15 and positiveness of *N*, we get

(67) $$\begin{array}{c} ɛ\left(1-\frac{N\left(x_{0}\right)}{K}\right)-a\frac{\phi \left(N\left(x_{0}\right)\right)}{N\left(x_{0}\right)}P\left(x_{0}\right)\geq 0. \end{array}$$

Hence, *N*(*x*_0_) < *K*. This in turn implies that $\max_{x\in \bar{\Omega }}N(x)\leq K$.

Analogously, by setting *ψ*_2_ = *D*_2_ *P*, we get from the second equation of system (64) that

(68) $$\begin{array}{c} \Delta \psi_{2}-M\left(P\right)P+b\phi \left(N\right)P=0,\;x\in \Omega ,\\ \frac{\partial \psi_{2}}{\partial \upsilon }=0,\;x\in \partial \Omega . \end{array}$$

Let $x_{0}\in \bar{\Omega }$ be such that $\psi_{2}(x_{0})=\max_{x\in \bar{\Omega }}\psi_{2}$. Then, by Lemma 15 and positiveness of *P*, we get

(69) $$\begin{array}{c} -M\left(P\left(x_{0}\right)\right)+b\phi \left(N\left(x_{0}\right)\right)\geq 0. \end{array}$$

That is,

(70) $$\begin{array}{c} -\frac{\gamma +\delta P\left(x_{0}\right)}{1+P\left(x_{0}\right)}+b\phi \left(N\left(x_{0}\right)\right)\geq 0. \end{array}$$

Hence,

(71) $$\begin{array}{c} \frac{\gamma +\delta P\left(x_{0}\right)}{1+P\left(x_{0}\right)}\leq b\phi \left(N\left(x_{0}\right)\right)\leq b\phi \left(K\right). \end{array}$$

So, *P*(*x*_0_) ≤ (*bϕ*(*K*) − *γ*)/(*δ* − *bϕ*(*K*)). This in turn implies that

(72) $$\begin{array}{c} \underset{x\in \bar{\Omega }}{\max}\;P\left(x\right)\leq \frac{b\phi \left(K\right)-\gamma }{\delta -b\phi \left(K\right)}. \end{array}$$

**Theorem 17**

Let *μ*_1_ be the smallest positive eigenvalue of the operator −Δ on *Ω* with the homogeneous Neumann boundary condition. If

(73) $$\begin{array}{c} \mu_{1}D_{1}>ɛ+\frac{aL}{2}+\left(a+\frac{b}{2}\right)K_{0}H,\\ \mu_{1}D_{2}>-\gamma +bL+\frac{aL}{2}+\frac{b}{2}K_{0}H, \end{array}$$

where *K*_0_ = (*bϕ*(*K*) − *γ*)/(*δ* − *bϕ*(*K*)) and *γ* < *bϕ*(*K*) < *δ*, then (64) has no nonconstant positive classical solution.

**Proof**

Assume that (*N*, *P*) is a nonconstant positive classical solution of (64). By (65), there exists a positive constant *C* = *C*(*K*, *b*, *γ*, *δ*) such that *N*(*x*), *P*(*x*) ≤ *C*. Let $\bar{N}=(1/|\Omega |)\int_{\Omega }Ndx$ and $\bar{P}=(1/|\Omega |)\int_{\Omega }P\;dx$. Multiplying the first equation of (64) by $N-\bar{N}$ and integrating over *Ω* by parts, we have

(74) D1∫Ω|∇(N−N¯)|2dx =∫Ω[ɛ(1−NK)N−aϕ(N)P](N−N¯)dx =∫Ω[ɛ(1−NK)N−aϕ(N)P−ɛ(1−N¯K)N¯+aϕ(N¯)P¯](N−N¯)dx =∫Ω[ɛ(N−N¯)−ɛK(N−N¯)(N+N¯)−aϕ(N)P+aϕ(N¯)P¯](N−N¯)dx =∫Ω[ɛ(1−1K(N+N¯))(N−N¯)2−a(ϕ(N)P−ϕ(N¯)P¯)(N−N¯)]dx ≤∫Ω[ɛ(N−N¯)2−a(ϕ(N)P−P¯ϕ(N)+P¯ϕ(N)−ϕ(N¯)P¯)(N−N¯)]dx ≤∫Ω[ɛ(N−N¯)2−a((P−P¯)ϕ(N)+P¯(ϕ(N)−ϕ(N¯)))×(N−N¯)]dx ≤∫Ω[ɛ(N−N¯)2+aL|P−P¯||N−N¯|+aK0H(N−N¯)2]dx ≤∫Ω[ɛ(N−N¯)2+aL((P−P¯)22+(N−N¯)22)+aK0H(N−N¯)2]dx =∫Ω[(ɛ+aL2+aK0H)(N−N¯)2+aL2(P−P¯)2]dx.

Similarly, we get

(75) D2∫Ω|∇(P−P¯)|2dx =∫Ω[−(δ+γ−δ1+P)P+bϕ(N)P](P−P¯)dx =∫Ω[−(δ+γ−δ1+P)P+bϕ(N)P+(δ+γ−δ1+P¯)P¯−bϕ(N¯)P¯](P−P¯)dx =∫Ω[−δ(P−P¯)−(γ−δ)(P1+P−P¯1+P¯)+b(ϕ(N)P−ϕ(N¯)P¯)](P−P¯)dx =∫Ω[−δ(P−P¯)+(δ−γ)P−P¯(1+P)(1+P¯)+b(ϕ(N)P−ϕ(N¯)P¯)](P−P¯)dx =∫Ω[(−δ+δ−γ(1+P)(1+P¯))(P−P¯)+b(ϕ(N)P−ϕ(N)P¯+ϕ(N)P¯−ϕ(N¯)P¯)]×(P−P¯)dx ≤∫Ω[−γ(P−P¯)2+b(ϕ(N)(P−P¯)+(ϕ(N)−ϕ(N¯))P¯)×(P−P¯)]dx ≤∫Ω[−γ(P−P¯)2+bL(P−P¯)2+bK0H|N−N¯||P−P¯|]dx ≤∫Ω[(−γ+bL)(P−P¯)2+bK0H(|N−N¯|22+|P−P¯|22)]dx =∫Ω[(−γ+bL+bK0H2)(P−P¯)2+bK0H20(N−N¯)2]dx.

Adding (74) and (75), we obtain

(76) ∫Ω[D1|∇(N−N¯)|2+D2|∇(P−P¯)|2]dx ≤∫Ω[(ɛ+aL2+(a+b2)K0H)(N−N¯)2+(−γ+bL+bK0H2+aL2)(P−P¯)2]dx.

By using the Poincare inequality (Theorem 11.11, page 112, in [15]), it follows that

(77) μ1∫Ω[D1(N−N¯)2+D2(P−P¯)2]dx ≤∫Ω[(ɛ+aL2+(a+b2)K0H)(N−N¯)2+(−γ+bL+bK0H2+aL2)(P−P¯)2]dx.

Taking into account (73), we obtain

(78) μ1∫Ω[D1(N−N¯)2+D2(P−P¯)2]dx ≥∫Ω[(ɛ+aL2+(a+b2)K0H)(N−N¯)2+(−γ+bL+bK0H2+aL2)(P−P¯)2]dx.

From (77) and (78), we get that $N=\bar{N}$ and $P=\bar{P}$, which is a contradiction. So, system (64) has no nonconstant positive classical solution. This completes the proof.

## 6. Conclusion and Discussion 

In this paper, we study a diffusive predator-prey model with nonconstant death rate and general nonlinear functional response. Firstly, stability analysis of the equilibrium for reduced ODE system is discussed. Secondly, sufficient and necessary conditions which guarantee the predator and the prey species to be permanent are obtained. Furthermore, sufficient conditions for the global asymptotical stability of the unique positive equilibrium of the system are derived by using the method of Lyapunov function. Finally, we show that there are no nontrivial steady state solutions for certain parameter configuration.

Note that hypotheses (i)–(v) in Section 1 are satisfied if function *aϕ*(*N*) represents Holling type II functional response or Holling type IV functional response; that is, *aϕ*(*N*) = *aN*/(*β* + *N*) or *aϕ*(*N*) = *aN*/(*β* + *N* ^2^). Hence, we can obtain the sufficient and necessary conditions which guaranting the predator and the prey species of the system with monotonic or nonmonotonic functional response to be permanent. If *ϕ*(*N*) = *N*/(*β* + *N*), system (7) reduces to system (1) that was studied by Duque and Lizana in [7]. It is shown that our result supplements and covers the main results of Duque and Lizana's paper [7].
